# Fundamental Understanding of Multicellular Triboelectric Nanogenerator with Different Electrical Configurations

**DOI:** 10.3390/mi14071333

**Published:** 2023-06-29

**Authors:** Zifan Li, Wee Chen Gan, Lihua Tang, Kean Chin Aw

**Affiliations:** 1 Department of Mechanical and Mechatronics Engineering, The University of Auckland, Auckland 1010, New Zealand; zli343@aucklanduni.ac.nz (Z.L.); l.tang@auckland.ac.nz (L.T.); 2 New Energy Science and Engineering, Xiamen University Malaysia, Sepang 43900, Malaysia; wcgan@xmu.edu.my

**Keywords:** triboelectric nanogenerator, multicellular, self-supporting structure

## Abstract

The single-cell triboelectric nanogenerator (TENG) often produces insufficient energy, leading to the use of a multicellular TENG structure. This work experimented with and simulated a dual-cell TENG with various configurations in parallel and series arrangements. The working principle of charge generation during each phase of a contact–separation cycle was explained through the analysis and comparison of five electrical configurations of a dual-cell TENG. Our observations indicate that measuring the output charge of a TENG provides a more reliable performance comparison. Finally, multicellular TENG with four cells arranged in an X-shape (X-TENG), self-supporting structure is fabricated and further experimented with, validating our conjectures derived from a dual-cell TENG.

## 1. Introduction

The advancement of technology increases the use of microelectronics, such as wearable sensors and the Internet of Things applications [1,2,3,4]. However, the power source for these devices still uses conventional energy storage, such as batteries, which are not sustainable and involve high maintenance costs [5,6,7]. Consequently, the demand for a sustainable power source has arisen [8]. In response, many alternative power supply methods were developed to harvest energy from the surroundings via photovoltaic [9,10,11], thermoelectric [12,13], piezoelectric [14,15,16], electromagnetic [17,18,19], and triboelectric [20,21] transductions. Compared to other methods, triboelectric nanogenerators (TENG) have demonstrated the most promising results thanks to their high efficiency at low frequencies [22], low fabrication costs [23], and the wide range of triboelectric materials available [24]. For the past decade, abundant work on the topic of TENG has been reported in the aspects of different working modes [25], modeling methods [26,27,28], structural designs [29,30], and applications [31].

The fundamental working principle of TENG is known as the triboelectric effect [32] and electrostatic induction. The triboelectric effect is a result of the difference in electron affinity between two materials. Electron affinity is associated with the energy required to acquire or lose an electron to a neutral atom or molecule to form a charged ion. When two materials with different electron affinities come into contact, contact electrification would occur between them. Charged particles at the surface of those materials will tend to transfer between the materials with different electron affinities. This process creates a charge transfer, with one material becoming positively charged and the other material becoming negatively charged. After contact electrification, a change in the physical distance between the two triboelectric materials (consequently, the change in capacitance) will induce a potential difference in the external circuit to generate electrical power.

Various methods have been adapted to improve the performance of TENG. Common approaches involve the surface charge injection [33], surface nanostructure modification [34,35,36], environment control [37], and the charge pump [38]. However, the power output of a single TENG cell is still limited [39], which leads to the development of multicellular TENG. By connecting multiple TENG cells together, the total power output can be easily increased [29,40,41,42]. Many researchers have used this method to increase the total power output, and some studies even introduced power management circuits to harvest as much power as possible [43,44]. However, there is still limited research analyzing the performance of a multicell TENG with different electrical connections, and the underlying working principle of multicell TENG is yet to be unveiled. In [45], the team studied the power output of a three-cell sliding TENG connected in parallel and series and discussed the voltage measurement distortion by the internal capacitance of the instrument, but the explanation of the charge transfer characteristics in those configurations was limited, and their exact working principle remains undiscussed. Similarly, He et al. [46] experimentally measured the output characteristics of multiple TENGs connected in series and parallel configurations and claimed that the parallel connection produces a higher power compared to the series connection. However, their conclusion contradicts that of [45], and the explanation was based on fitting the experimental data, which is insufficient to explain the multicellular TENG working principle.

In this work, we fabricate a dual-cell TENG and experiment as well as simulate its performance with respect to a single-cell TENG, with various configurations in parallel and series arrangements. Through the analysis and comparison of five electrical configurations of a dual-cell TENG, we qualitatively explain the working principle of energy generation for each configuration by analyzing the charge transfer within the structure during each phase of a contact–separation cycle. Finally, a self-supporting structure with four TENG cells arranged in an X-shape (X-TENG) is fabricated and further experimented to demonstrate the validity of our conjectures derived from a dual-cell TENG.

## 2. Experiment and Finite Element Simulation

This section describes the definitions of different configurations, the experimental setup, and the finite element simulation model for a dual-cell TENG. The initial experimental result for a single TENG cell is used as a reference to compare against various configurations in the later sections.

### 2.1. TENG Configuration and Experimental Setup

**Figure 1:**
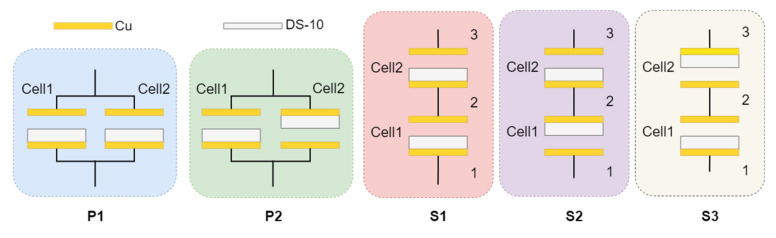
Illustration of the five connection configurations tested.

Two single parallel-plate TENG cells were fabricated first using copper tape and a Dragon skin-10 (DS-10) silicone elastomer, with copper being the positive triboelectric material and DS-10 being the negative triboelectric material (see Figure S1 in the Supplementary Materials). The positive triboelectric side of the cells was formed by cutting the copper tape to the right size, and the negative triboelectric side was fabricated through three steps. First, a mold was created using 3D printing, with the edges raised precisely 1 mm. Next, DS-10 was carefully mixed and applied to the mold to cure. After the curing process, the copper tape was applied to the back of the DS-10 to complete the cell. The structure of the TENG cell followed the typical metal–dielectric TENG configuration, and the energy output was generated through a repetitive contact–separation motion of the two triboelectric materials. With the characterized output of single TENG cells, the performance of a multicell TENG was demonstrated using the two fabricated TENG cells (Cell 1 and Cell 2), which can be arranged with five different electrical configurations (P1, P2, S1, S2, and S3), covering most possible scenarios of connecting two TENG cells, as shown in Figure 1. P1 and P2 are parallel configurations and S1, S2, and S3 are series configurations, with different arrangements of the positive and negative triboelectric layers and electrodes.

Note that in S1–S3 configurations, electrical nodes associated with the cells were labeled as 1, 2, and 3 (Figure 1). When measuring across nodes 1 to 3, the electrometer measured the combined output from both Cell 1 and Cell 2. Also note that in this article, the contact–separation cycle of the TENG in both cells was synchronized, and the contact forces for both cells were also kept constant, which was achieved by placing both cells the same distance away from the center of the impact plate and the stationary plate (see Figure S3 in the Supplementary Materials). It is important to realize that in the real-life application of multicellular TENG structures, the contact–separation cycles of the cells could be out of sync with different contact forces and could impact the overall power output, which, however, is out of the scope of this work.

**Figure 2:**
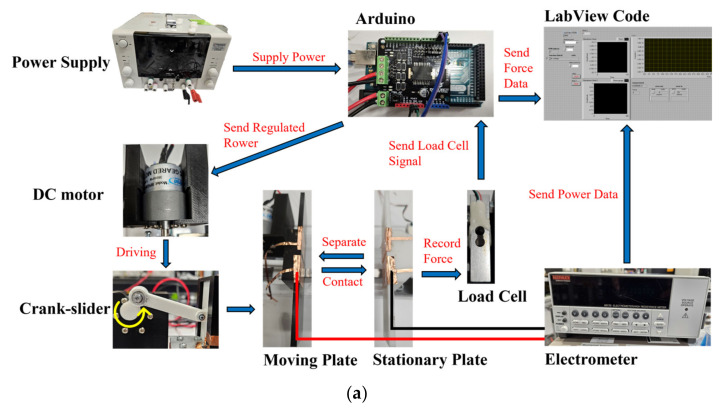

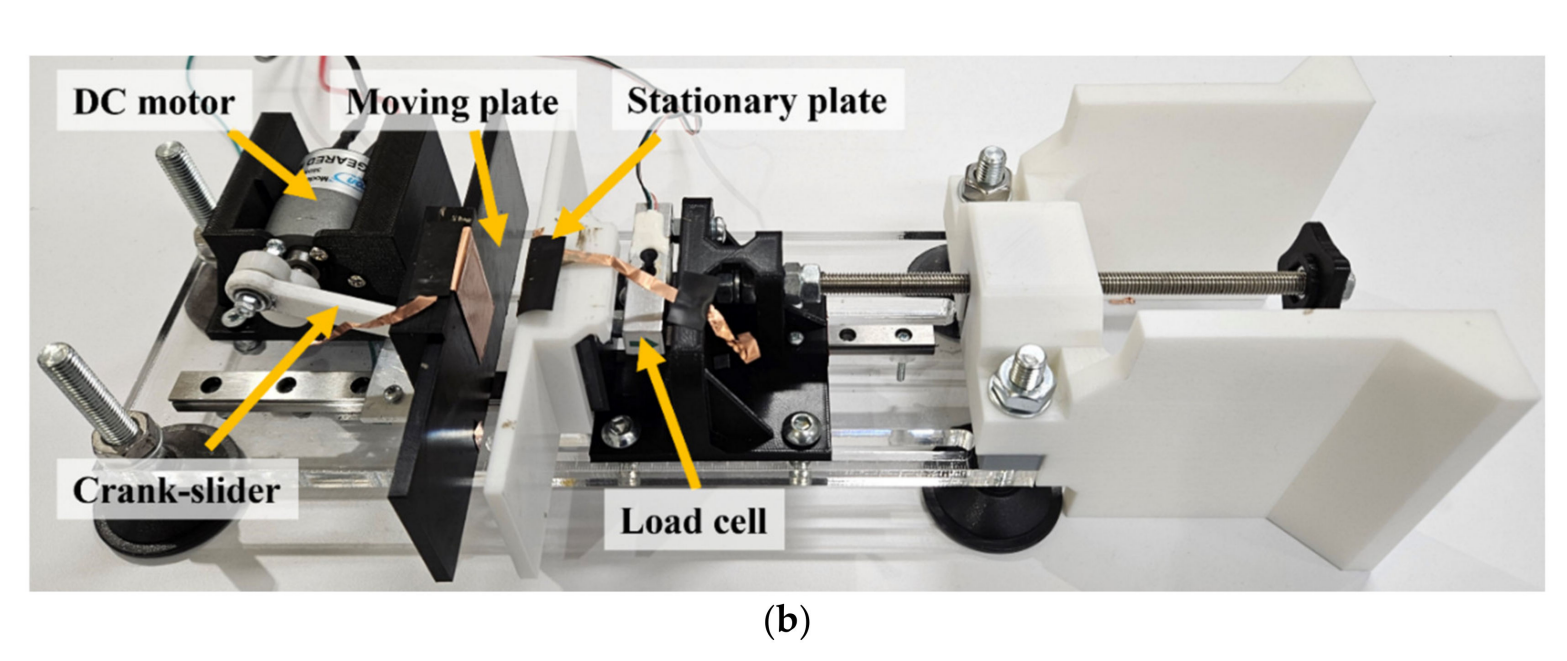
(**a**) Flow chart illustrating the experimental setup and (**b**) the testing rig used to characterize the performance of the different configurations.

The overall experimental setup is shown in Figure 2a. A customized driving system (Figure 2b) was built with a crank-slider mechanism and a DC motor controlled by an Arduino board. The dual-cell TENG was fixed on the stationary plate and the moving plate impacted the TENG at a certain amplitude of force, which was measured through the load cell. The electrical outputs (the peak-to-peak open-circuit voltage (V_OC_) and the short-circuit charge transfer (Q_SC_)) from TENG were measured by the electrometer (Keithley, Model: 6517B) and processed in LabVIEW.

### 2.2. Finite Element Simulation

A finite element simulation model was also built using COMSOL Multiphysics to compare with the experiment and assist in the understanding of the working principle of the dual-cell TENG with different electrical configurations. The parameters of a single-cell TENG are listed in Table 1.

**Figure 3:**
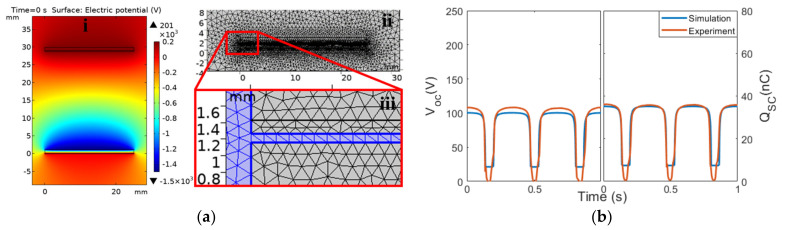
(**a**) Finite element model and mesh in COMSOL for a TENG cell and (**b**) electrical outputs of a single TENG cell from the simulation and the experiment.

A 355 pF capacitance was added in the simulation to represent the overall capacitance of the measurement instrument [45,47]. The V_OC_ and Q_SC_ of Cell 1 obtained from the simulation model (Figure 3a(i)) were compared with the results acquired experimentally, as shown in Figure 3b. In general, both the experiment and the simulation yielded around 100 V and 34.5 nC. The non-zero V_OC_ response when the TENG was fully compressed in the simulation was due to the fact that the COMSOL simulation could not simulate a true contact, and a small gap was required for meshing between the two materials, as illustrated in Figure 3a(ii,iii) is the magnified view. Nevertheless, as we were more interested in the dual-cell system and the performance difference between the configurations, this discrepancy was acceptable. With the validated results, the performance with different electrical configurations could then be analyzed in the following sections.

## 3. Results and Discussion

In this section, the electrical outputs from the experiment and the simulation of the dual-cell TENG with all configurations are presented. By inspecting the charge transfer within the structure during each phase of the contact–separation cycle, we aim to explain the working principle of the TENG in different electrical configurations.

### 3.1. Configuration P1

**Figure 4:**
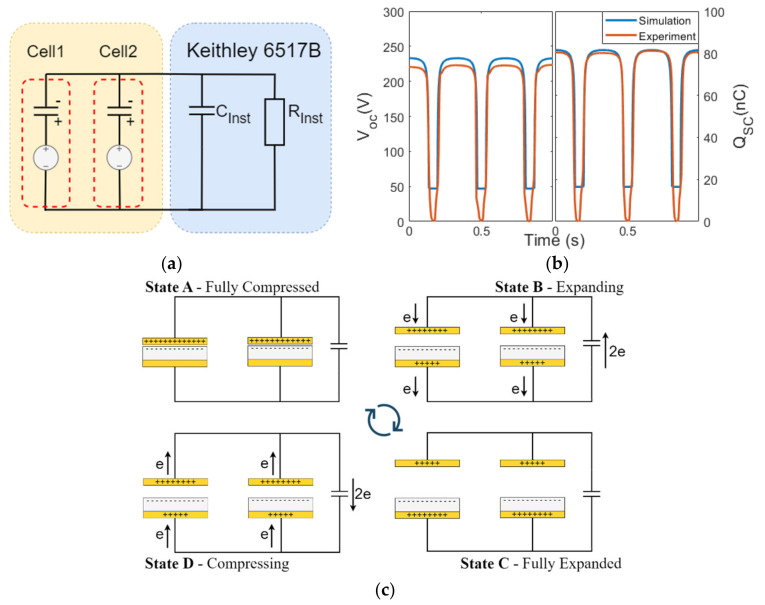
(**a**) Circuit representation of two TENG cells in configuration P1 connected to the electrometer. (**b**) Electrical outputs from the simulation and the experiment of configuration P1. (**c**) Charge transfer in the contact–separation cycle of the P1 configuration.

The results when the cells were arranged in configuration P1 are presented in this section. In this configuration, the positive triboelectric layers of both cells were connected, and similarly for the negative triboelectric layers in both cells. The measurement instrument (Keithley 6517b electrometer) contains an internal capacitance of C_inst_ = 355 pF and internal resistance of 200 TΩ, which were connected in parallel to the two TENG cells (Figure 4a) and taken into account in the simulation. The simulated and measured maximum V_OC_ were 232 V and 223 V, respectively, and Q_SC_ was 81.8 nC and 81.6 nC, respectively, as shown in Figure 4b. Note that when using an oscilloscope to measure the voltage, a typical oscilloscope probe has an input capacitance of 10 pF and an input resistance of 1 MΩ or 10 MΩ, which will produce a different voltage reading compared to the Keithley electrometer.

As compared to the results of the single-cell TENG (Figure 3b), the V_OC_ and Q_SC_ for the P1 configuration doubled. The related charge transfer is explained in Figure 4c (note that the use of “e” in the context of this manuscript represents the negative charges). Suppose that each TENG cell contributes a charge of “e”. When the two cells were connected in configuration P1, the amount of charge flowing into the load was “2e = e + e” (Kirchhoff’s current law), aligning with the doubled Q_SC_ that was measured. The V_OC_ was also doubled compared to that of a single TENG cell, which is different from our understanding about the voltage measured for two parallel capacitors or batteries. This effect was due to the C_inst_ and can be explained using Q_SC_ = C V_OC_, where Q_SC_ is the charge contributed because of “2e”. As the instrumental resistance, R_inst_, was extremely large (200 TΩ), the instrumental load was equivalent to a capacitive load, with C_inst_ = 355 pF, which was also much larger than the capacitance of Cell 1 and Cell 2, leading to the measured output voltage, V_OC_, being proportional to the charge transferred from the TENG (see Figure S2 in the Supplementary Materials). During the contact–separation cycle, the charge transferred through the load (States B and D) would be a combined effort from both cells, doubling the measured Q_SC_ transferred as well as the V_OC_ generated by a single cell.

### 3.2. Configuration P2

**Figure 5:**
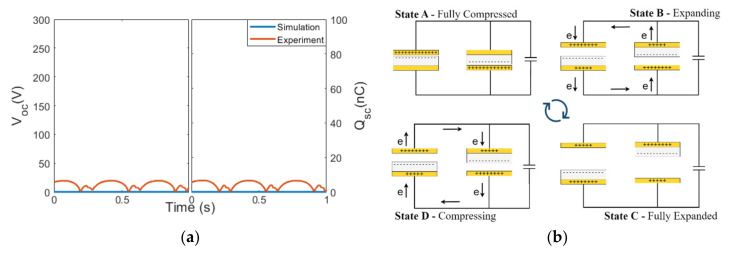
(**a**) Electrical outputs from the simulation and the experiment of configuration P2. (**b**) Charge transfer in the contact–separation cycle of configuration P2.

In contrast to configuration P1, configuration P2 displayed an extremely low output, with a V_OC_ of 33.51 V and a Q_SC_ of 12 nC from the experiment, and no output at all from the simulation (Figure 5a). When the two cells were connected in configuration P2, most charge transfer occurred between the negative triboelectric layer of Cell 1 to the positive triboelectric layer of Cell 2, flowing back and forth inside the two cells instead of through the load, which would theoretically result in a zero-charge transferred through the load, as indicated in Figure 5b. However, Cell 1 and Cell 2 were not perfectly identical in the experiment, causing some charge to leak into the load and resulting in a small V_OC_ and Q_SC_ (see Figure S4 in the Supplementary Materials).

### 3.3. Configuration S1

**Figure 6:**
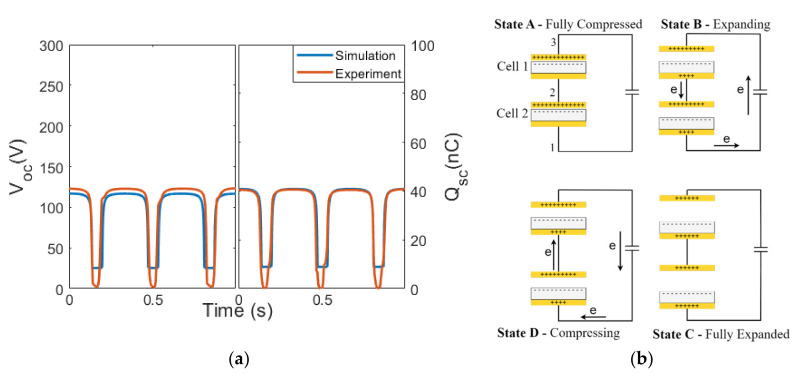
(**a**) Electrical outputs from the simulation and the experiment of configuration S1. (**b**) Charge transfer in the contact–separation cycle of configuration S1.

The results of the S1 configuration showed that the voltage output (V_OC_ = 124.3 V) was comparable to that of one single TENG cell. The total Q_SC_ through the load remained unchanged, reaching 40.2 nC. In configuration S1, the load was connected across nodes 3 and 1 (Figure 6b), which are the positive triboelectric layer of Cell 2 (node 3) and the negative tribo-layer of Cell 1 (node 1), while node 2, i.e., the positive triboelectric layer of Cell 1, was directly connected to the negative triboelectric layer of Cell 2. This is an analogy to two batteries connected in series. As both cells underwent contact–separation (States B or D), the charge transferred out from node 3 to the load and then back to node 1 was of a similar amount as a single TENG cell, which was “e”. The same amount of charge “e” was also internally redistributed in the two cells. Again, due to the large instrumental capacitance, C_inst_, which by Q_SC_ = C V_OC_, this explains why V_OC_ was barely changed compared to the single cell of TENG, since the transferred charge was similar. This caused the difference in the typical two batteries in series, which doubled the V_OC_ of a single battery.

### 3.4. Configurations S2 and S3

**Figure 7:**
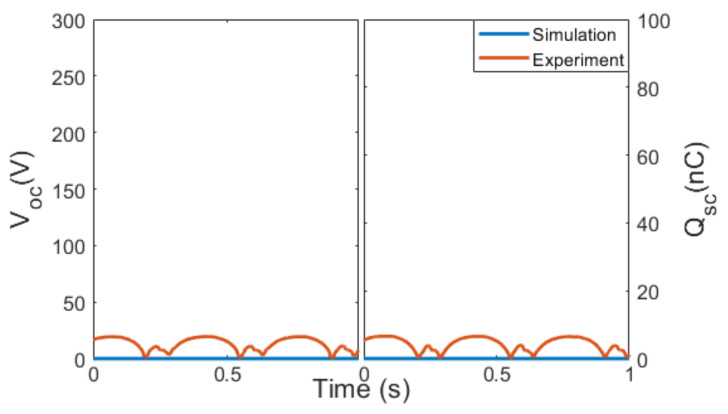
Electrical outputs from the simulation and the experiment of configuration S2 (or S3).

When the two TENG cells were connected in either configuration S2 or S3, the V_OC_ and Q_SC_ measured with the load connected between nodes 1 and 3 were only 18.4 V and 6 nC, respectively (Figure 7). As the configuration was analogous to the two batteries connected in series, opposingly, there was no charge transfer through the load, and the V_OC_ and Q_SC_ should be theoretically equal to zero. However, as aforementioned, Cell 1 and Cell 2 were not perfectly identical, leading to small V_OC_ and Q_SC_ values.

### 3.5. Special Cases

**Figure 8:**
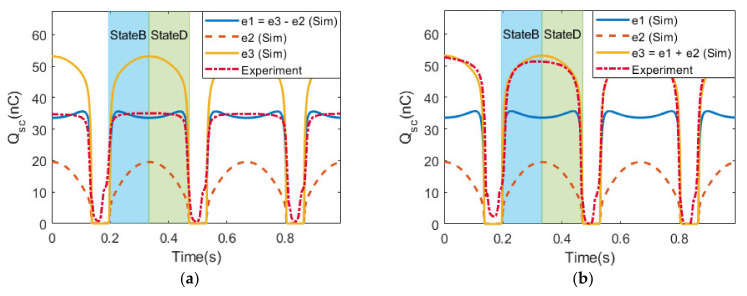

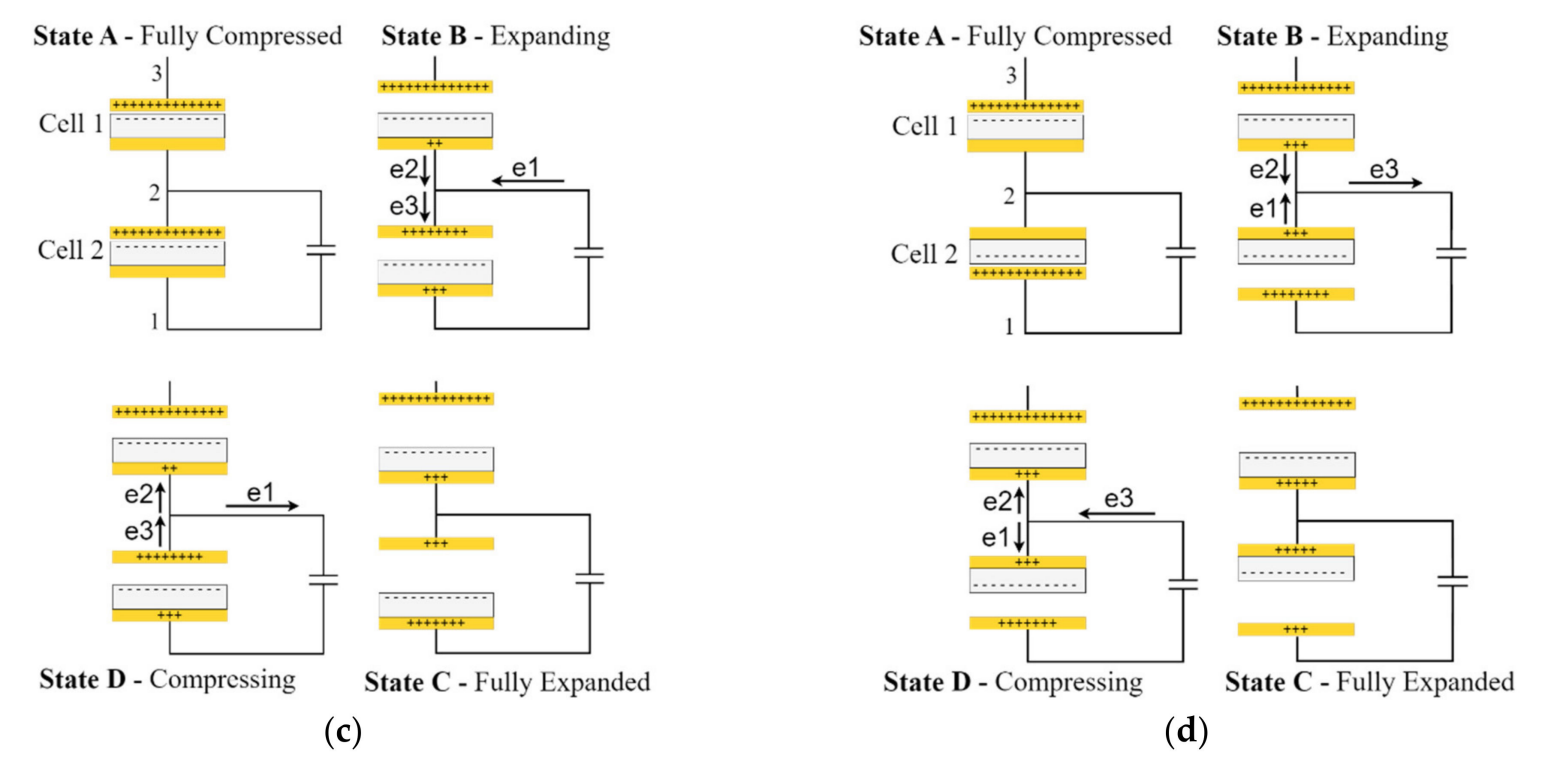
Simulation and experimental results of the magnitude of charge transfer through node 2 in (**a**) configuration S1-1 and (**b**) configuration S2-1. Charge transfer in the contact–separation cycle of (**c**) configuration S1-1 and (**d**) configuration S2-1.

To further elucidate the charge transfer mechanism, the V_OC_ and Q_SC_ were measured across only one TENG cell (nodes 1 and 2, while node 3 was disconnected) in a series configuration. The configuration in Figure 8c is similar to configuration S1 but the load was connected across Cell 1 (nodes 1 and 2), which was termed configuration S1-1 to differentiate it from configuration S1 in Section 3.3. Further, the configuration in Figure 8d, similar to configuration S2 in Section 3.4, was termed configuration S2-1. Configurations S1-1 and S2-1 can be treated similarly to connecting two batteries in series, aiding (the cathode of one battery was shorted with the anode of another battery) and opposing (two batteries were shorted via the same polarity), respectively, with one end of the terminal not connected to the load.

In configuration S1-1, V_OC_ and Q_SC_ reached 102.1 V and 34.5 nC, respectively, which was close to the performance of a single cell acting alone, with a slightly different output profile (Figure 8a). On the other hand, in configuration S2-1, V_OC_ and Q_SC_ reached 142 V and 47.3 nC, which was significantly higher than the single-cell output (Figure 8b). Such observation implies that even though Cell 2 was not directly involved, its existence could still impact the output when the load was only connected across Cell 1. The difference in output between configurations S1-1 and S2-1 can be explained using the conservation of charge around node 2. In configuration S1-1, during State B, most charge (e1) transferred from the load into node 2, but a small amount of charge (e2) flowed from Cell 1 into node 2, achieving electrostatic equilibrium, such that e1 = e3 − e2 (Figure 8c). In State C, all charges achieved the equilibrium, and there was no current flow in the load. In State D, the charge flow about node 2 was reversed to achieve electrostatic equilibrium again. In contrast, configuration S2-1 yielded a different result than configuration S1-1 because at node 2, the triboelectric layers of Cells 1 and 2 were of a similar triboelectric affinity/polarity. During State B (Figure 8d), the charge from both Cells 1 and 2 (e2 and e1) flowed out into node 2 and then flowed out into the load as e3 = e1 + e2. Similarly, in State D, the amount of charge, e3, flowed back into node 2 to reach electrostatic equilibrium. The experimentally measured transferred charge supports our conjectures. In this scenario, Cell 1 behaved as a charge-enhancing layer by contributing additional charge, e2, flowing into the load to yield higher V_OC_ and Q_SC_ values.

### 3.6. Comparison and Discussion

**Figure 9:**
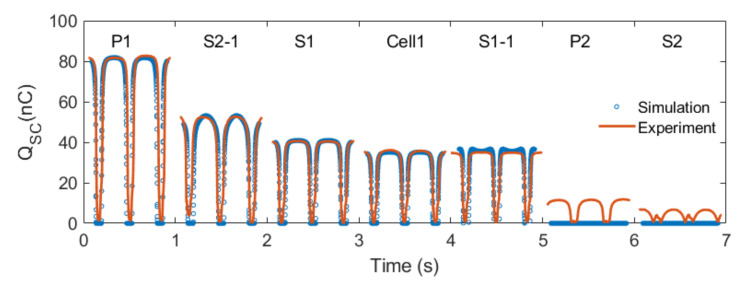
Simulation and experimental results of the magnitude of charge transfer for all configurations.

Results of different configurations of a dual-cell TENG are further demonstrated in Figure 9. It is noted that configuration P1 produced the highest Qsc, doubling that of a single TENG cell. Configuration S2-1 was the second choice, with a much higher charge transfer compared to a single cell. Configurations S1 and S1-1 did not show any significant increase as compared to a single TENG cell, and configurations P2, S2, and S3 canceled out the charge transfer from two cells, leading to theoretically no output.

In addition, the conjectures about the charge transfer seemed to successfully explain the performance of a dual-cell TENG structure in all sorts of configurations. To further confirm our conjectures, a further experiment was carried out for TENGs made from fluorinated ethylene propylene (FEP) and DS-10, which formed a dielectric–dielectric TENG. The fabrication process for DS-10/FEP TENG was mostly the same compared to the DS-10/Cu TENG mentioned previously, with the only difference being an extra layer of FEP sheet applied to the Cu layer (Figure S5 in the Supplementary Materials). Results similar to those of Figure 9 were observed (Figure S6 in the Supplementary Materials).

## 4. Further Case Study

**Figure 10:**
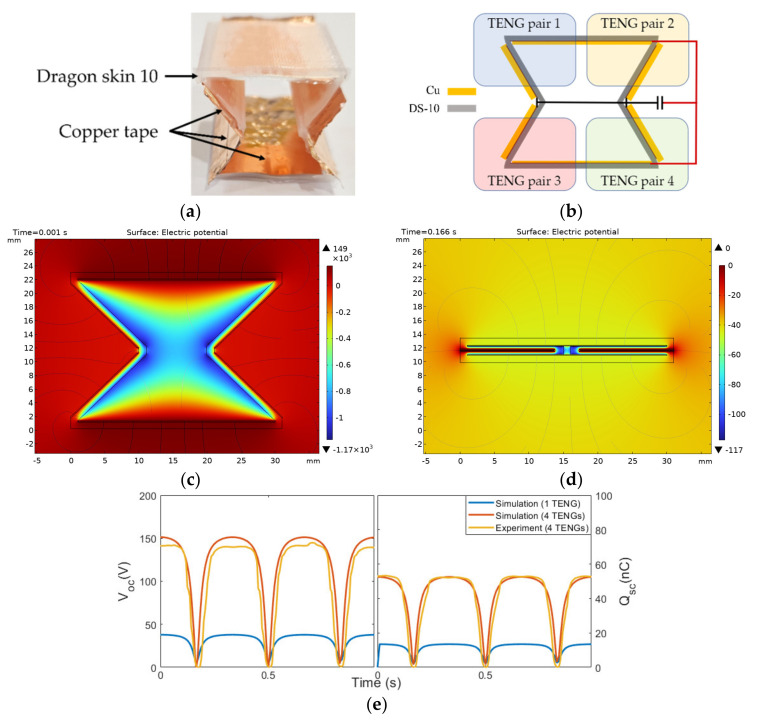
(**a**) Prototype of the X-TENG. (**b**) Wiring between cells of the X-TENG. (**c**,**d**) The electrical potential contours of the X-TENG from the finite element simulation in fully expanded and compressed states. (**e**) Comparison of electrical outputs from the finite element simulation and the experiment.

Based on our finding about the dual-cell TENG with a parallel configuration that maximized the charge transfer, we implemented a multicellular TENG based on the X-TENG structure [48,49] using Cu and DS-10 (Figure 10a). The X-shaped TENG consisted of four cells of TENG connected in configuration P1 (Figure 10b). A finite element model using COMSOL was also established using the parameters listed in Table 1, and the electric potential contours in expanded and compressed states are shown in Figure 10c,d. Each cell of TENG had a surface area of about 3.7 cm^2^, which was slightly smaller than half the size of the parallel-plate dual-cell TENG used in the previous sections. Both the simulation and experimental results indicated that the output of an X-TENG can produce over 145 V and 57 nC (Figure 10e), which is about 4 times that of a single-cell X-TENG. The results further confirmed that the P1 configuration was the most effective configuration in a multicellular TENG.

## 5. Conclusions

In conclusion, we thoroughly studied the output of a dual-cell TENG with different electrical configurations. Five configurations and a few special cases were studied, and the working principle was explained based on the charge transfer obtained from the finite element simulation and the experiment. As expected, both the experiment and simulation indicated that configuration P1 was the most effective way to connect two cells, almost doubling the output from a single cell, and an explanation of this occurrence was provided in detail. Our observations indicated that measuring the output charge of a TENG provides a more reliable performance comparison than the output voltage due to the varying capacitance and impedance values of different measurement instruments. The results also revealed that an additional TENG cell, though not connected to the load circuit, still enhanced the output with configuration S2-1, and this was explained. Finally, a compact, self-supporting structure that contains four TENGs (X-TENG) was fabricated to demonstrate a compact, multicellular TENG connected in configuration P1, to increase its electrical output. Future work will focus on analyzing the performance of the multicellular TENG with a non-synchronized contact–separation cycle for the cells, which is often a more realistic situation in practice.

## Figures and Tables

**Table 1:** Simulation parameters of a single-cell TENG.

Property	Value
Positive triboelectric material	Cu
Thickness of positive triboelectric layer	d_1_ = 0.16 mm
Negative triboelectric material	Silicone
Thickness of negative triboelectric layer	d_2_ = 1 mm
Permittivity of negative triboelectric layer	ϵ_r_ = 3.53
Triboelectric surface area	A = 7.5 cm^2^
Initial surface charge density	4.8 nC/cm^2^
Maximum separation gap	X_max_ = 2.77 cm

## Data Availability

Not applicable.

## Supplementary Materials

The following supporting information can be downloaded at: https://www.mdpi.com/article/10.3390/mi14071333/s1, Figure S1: A photo of the parallel plate TENG used in the experiment. Figure S2: Charge output versus voltage output measured using Keithely 6517B electrometer for different configurations. Figure S3: Photos of the dual cells in (a) fully separated state and (b) fully closed state in synchronized motion. Both cell are placed same distance away from the center of the plate to achieve even exitation force. Figure S4: VOC and QSC of Cell 1 and Cell 2 measured in experiment. Figure S5: Illustration for DS-10/FEP TENG cell structure. Figure S6: (a) Peak-to-peak open circuit voltage and (b) Short-circuit charge transfer for all configurations of TENG made from FEP and DS-10.
