# Supplementary material for: Fundamental Understanding of Multicellular Triboelectric Nanogenerator with Different Electrical Configurations
Source: Micromachines (Basel). 2023 Jun 29;14(7):1333. doi: 10.3390/mi14071333 (PMC10383503; doi:10.3390/mi14071333)
Supplement: Supplementary file 1 [file micromachines-14-01333-s001.zip › micromachines-2450498-supplementary.pdf]

Supplementary Materials

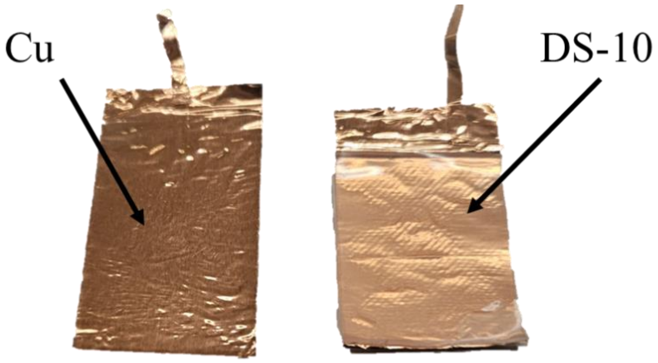

Figure S1. A photo of the parallel plate TENG used in the experiment

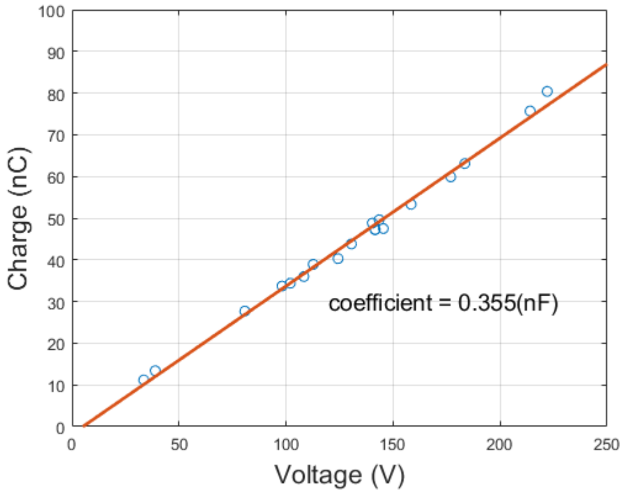

Figure S2. Charge output versus voltage output measured using Keithely 6517B electrometer for different configurations

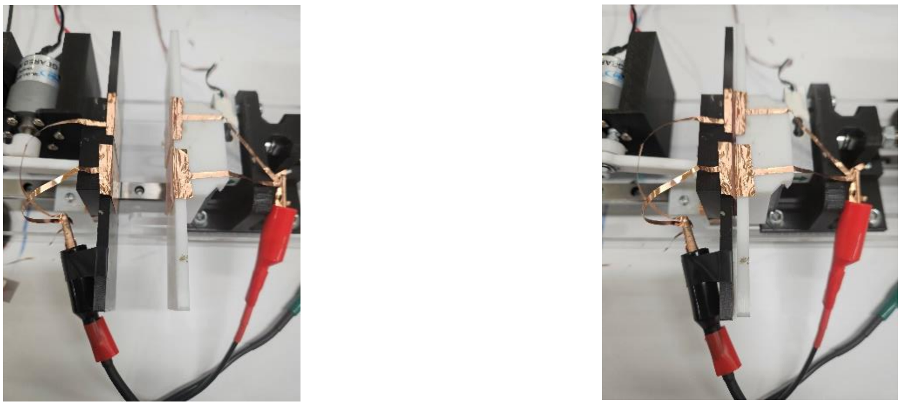

(a) (b)

Figure S3. Photos of the dual cells in (a) fully separated state and (b) fully closed state in synchronized motion. Both cell are placed same distance away from the center of the plate to achieve even exitation force

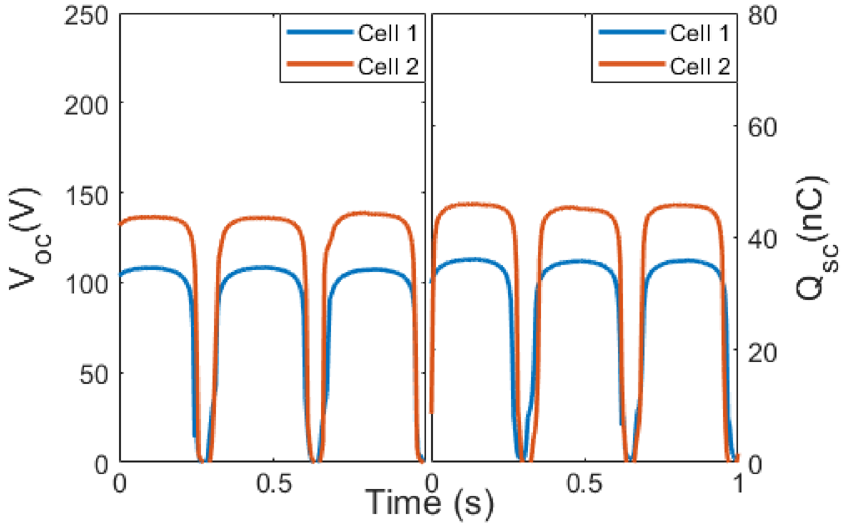

Figure S4. V_{OC} and Q_{SC} of Cell 1 and Cell 2 measured in experiment.

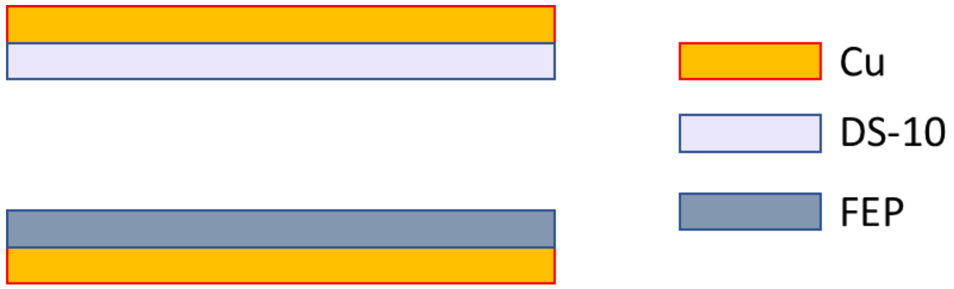

Figure S5: Illustration for DS-10/FEP TENG cell structure

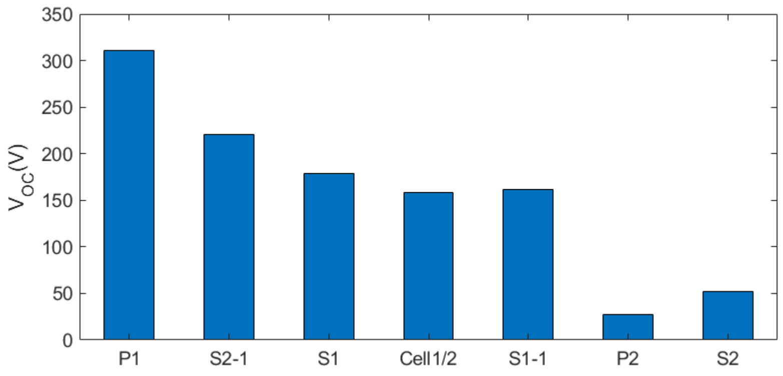

(a)

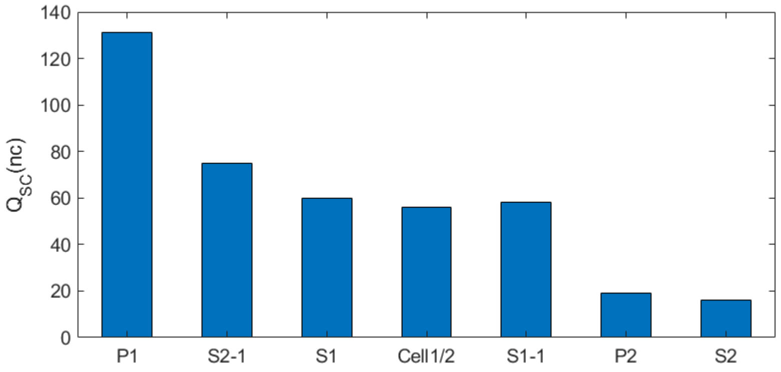

(b)

Figure S6. (a) Peak-to-peak open circuit voltage and (b) Short-circuit charge transfer for all configurations of TENG made from FEP and DS-10.
